# Increased susceptibility to pneumonia due to tumour necrosis factor inhibition and prospective immune system rescue *via* immunotherapy

**DOI:** 10.3389/fcimb.2022.980868

**Published:** 2022-09-07

**Authors:** Ryan Ha, Yoav Keynan, Zulma Vanessa Rueda

**Affiliations:** ^1^ Department of Medical Microbiology and Infectious Diseases, University of Manitoba, Winnipeg, MB, Canada; ^2^ Department of Community-Health Sciences, University of Manitoba, Winnipeg, MB, Canada; ^3^ Facultad de Medicina, Universidad Pontificia Bolivariana, Medellin, Colombia

**Keywords:** Tumour necrosis factor (TNF) inhibitors, immunotherapy, pneumonia, infection, opportunistic pathogens, cytokines, immunomodulation

## Abstract

Immunomodulators such as tumour necrosis factor (TNF) inhibitors are used to treat autoimmune conditions by reducing the magnitude of the innate immune response. Dampened innate responses pose an increased risk of new infections by opportunistic pathogens and reactivation of pre-existing latent infections. The alteration in immune response predisposes to increased severity of infections. TNF inhibitors are used to treat autoimmune conditions such as rheumatoid arthritis, juvenile arthritis, psoriatic arthritis, transplant recipients, and inflammatory bowel disease. The efficacies of immunomodulators are shown to be varied, even among those that target the same pathways. Monoclonal antibody-based TNF inhibitors have been shown to induce stronger immunosuppression when compared to their receptor-based counterparts. The variability in activity also translates to differences in risk for infection, moreover, parallel, or sequential use of immunosuppressive drugs and corticosteroids makes it difficult to accurately attribute the risk of infection to a single immunomodulatory drug. Among recipients of TNF inhibitors, *Mycobacterium tuberculosis* has been shown to be responsible for 12.5-59% of all infections; *Pneumocystis jirovecii* has been responsible for 20% of all non-viral infections; and *Legionella pneumophila* infections occur at 13-21 times the rate of the general population. This review will outline the mechanism of immune modulation caused by TNF inhibitors and how they predispose to infection with a focus on *Mycobacterium tuberculosis*, *Legionella pneumophila*, and *Pneumocystis jirovecii*. This review will then explore and evaluate how other immunomodulators and host-directed treatments influence these infections and the severity of the resulting infection to mitigate or treat TNF inhibitor-associated infections alongside antibiotics.

## 1 Introduction

Immunomodulators are compounds that can be used to either suppress or enhance the production or activity of immune factors such as antibodies, cytokines, chemokines, and immune cells. Some examples of these are tumour necrosis factor (TNF) antagonists, corticosteroids, interleukin (IL) supplements, and cytokine antagonists. Immunomodulators have found applications in the treatment of various autoimmune disorders, often targeting specific aspects of the immune system while not directly influencing others to minimize collateral damage. The altered immune response caused by immunomodulators may increase susceptibility to infectious diseases, particularly those caused by commonly encountered respiratory pathogens (Saravolatz et al., 1979; Wallis et al., 2004; Baddley et al., 2014; Murdaca et al., 2019; Chiu and Chen, 2020). The outcomes of infections in individuals treated with immunomodulators vary depending on the immunological mediators affected and the pathogen in question. With many pathogens exploiting the immune system to aid in their pathogenesis, it is crucial to employ the appropriate drug to achieve control of the underlying immune disease while recognizing and controlling specific infections associated with the administration of the immune-modulating agent.

Among immunomodulators, TNF inhibitors are widely used as treatment for a variety of conditions involving hyperactive immune disorders such as rheumatoid arthritis, psoriasis, and inflammatory bowel disease (IBD). Corticosteroids are often prescribed for the same diseases as TNF inhibitors; however, short courses are favored due to long-term consequences associated with corticosteroid administration. Compared to corticosteroids, TNF inhibitors have a lower risk profile, while also being more efficacious (Helliwell et al., 2008; Murdaca et al., 2019; Penso et al., 2021). Consequently, this increased immunosuppression comes at the cost of increased susceptibility to infection. The mechanisms behind inflammation in conditions treated with TNF blockade involve the same pathways used in response to intracellular pathogens, causing increased susceptibility and severity of pneumonia in people living with these diseases (Wallis et al., 2004; Curtis et al., 2011). Thus, special caution must be taken when administering TNF inhibitors to elderly individuals and individuals living in, or from areas of endemic tuberculosis (TB) disease or other pathogens that cause pneumonia.

The purpose of this review is to summarize the differences between TNF inhibitors and therapeutic immunomodulation on predisposition to pneumonia caused by select intracellular pathogens. Then, potential host-directed treatments that could mitigate or treat TNF inhibitor-associated infections alongside antibiotics will be reviewed with a focus on metformin.

Metformin will be discussed because of its status as an FDA-approved drug, its lower risk profile (including infection risk) with and without diabetes, evidence suggesting its potential as immunotherapy in the context of infections, and evidence of it ameliorating conditions treated with TNF inhibitors, making it an attractive drug for study (Howlett and Bailey, 1999; Kang et al., 2013; Son et al., 2014; Provinciali et al., 2015; Malik et al., 2018; Routy et al., 2019; Scheen, 2020; Shikuma et al., 2020; Chew et al., 2021; Li et al., 2021; Planas et al., 2021; Samuel et al., 2021). While traditionally used for increasing insulin sensitivity in people affected by diabetes, the precise mechanisms of metformin are not yet fully understood, but there exists evidence of therapeutic effect in various conditions not currently treated with metformin (Kang et al., 2013; Podhorecka et al., 2017; Ala and Ala, 2021; Kim et al., 2022). Among these effects is metformin’s ability to induce a mild immunosuppression, especially when compared to that of TNF inhibitors. This allows for finer modulation in individuals with hyperactive immune systems to enable persistence at a level in which it can preserve some degree of the immune response to circumvent the notable infection risk imposed by TNF inhibitor usage. Metformin-based treatments can possibly be used to restore the immune system to be equivalent to one that would resemble that of healthy individuals. Beyond the decreased risk of infection in individuals that take metformin, there is even evidence suggesting that metformin can be used as immunotherapy to treat infections, which will be further discussed.

In addition to drugs like metformin, cytokines can be supplemented or neutralized to control infections by manipulating the immune response in a way that is less permissive to pathogen growth. Some of these potential interventions will be discussed based on cytokine profiles and drug interventions from studies examining the host immune response in individuals, animal models, and cells infected with *Mycobacterium tuberculosis*, *Legionella pneumophila*, and *Pneumocystis jirovecii*.

### 1.1 TNF inhibitors’ impact on risk of infection

Due to their immunosuppressive functions, individuals taking TNF inhibitors are at a heightened risk of infection and at risk for a more severe course compared to healthy individuals not taking immunosuppressants, with up to 14% of individuals discontinuing TNF inhibitor treatment due to infections (Stober et al., 2018). This is especially true for respiratory pathogens such as *Mycobacterium* spp., *Legionella* spp., and *Pneumocystis jirovecii* due to the high prevalence of latent infections and wide geographical distribution. The infectious risks of TNF inhibitors are further increased in regions of the world where these pathogens are endemic, particularly among persons from countries or regions of highly prevalent TB (World Health Organization; Wallis et al., 2004). However, with approximately 50 individuals affected by rheumatoid arthritis per 100,000 inhabitants globally and TNF inhibitors comprising the most frequently prescribed immune modulators, this burden to diligently monitor patients who are either slated to take TNF inhibitors or already taking them extends beyond individuals from high-risk countries (Almutairi et al., 2021). Canada also has one of the highest incidences of IBD in the world, ranging from 18.7 per 100,000 to 54.6 per 100,000 based on province and some reports suggesting up to 319 per 100,000 across Canada for Crohn’s disease alone (Ng et al., 2017; Kaplan et al., 2019). Global incidence of IBD is predicted to increase, with figures rising across the globe, even in regions that traditionally had lower incidence (Ng et al., 2017). Cumulatively, the increase in the need for TNF inhibitors and increased globalization will produce a setting where TNF inhibitor-associated infections is a growing issue that must be addressed.

## 2 TNF inhibitors

TNF-α is a proinflammatory cytokine involved in early recruitment and activation of the innate immune system in response to infection. It is produced by a myriad of cell types to induce differentiation and activation of phagocytes, inflammation in endothelial cells, and apoptosis of infected cells, making it an essential mediator in the immediate immune response by kickstarting much of the production and expansion of immune factors while inhibiting regulatory immune cells such as Tregs (Shibuya et al., 1998; Sedger and McDermott, 2014). As such, it is a critical link between the innate and adaptive immune systems. Due to its central role in the early innate immune response, TNF inhibition results in an increase in susceptibility to infection (Minozzi et al., 2016; Blanchard et al., 2017; Murdaca et al., 2019). While TNF inhibitors share a target pathway they differ in structure, distribution, efficacy, specificity, conditions treated, administration route, and even risks (Scallon et al., 2002; Palframan et al., 2009; Suzuki et al., 2010; Biancheri et al., 2015; Mitoma et al., 2018; Gerriets et al., 2021). The differences between the TNF inhibitors covered in this review can be found summarized in **Table 1**.

**Table 1:** Summary of tumour necrosis factor (TNF) antagonists.

TNF antagonist	Structure^a,b,c,d^	Binding target^a,c,e,f,g^	Half-life^a,c,d,f,h^	Risk of infection^i,j,k,l,m,n^
**Adalimumab**	Humanized mAb	Monomeric and trimeric sTNF-α and tmTNF-α	10-20 days	++
**Infliximab**	Chimeric mAb (murine Fab, human Fc)	Monomeric and trimeric sTNF-α and tmTNF-α	8-10 days	+++
**Etanercept**	Human Fc-TNF-R fusion protein	Trimeric sTNF-α and lymphotoxin	3-5.5 days	+
**Golimumab**	Humanized mAb	Monomeric and trimeric sTNF-α and tmTNF-α	9-15 days	N/A
**Certolizumab**	PEGylated human Fab	Monomeric and trimeric sTNF-α and tmTNF-α	14 days	N/A

mAb, monoclonal antibody; sTNF-a, soluble TNF-a; tmTNF-a, transmembrane TNF-a; Fab, antigen-binding fragment; Fc, constant fragment; TNF-R, TNF-receptor; PEG, polyethylene glycol.. ^a^Weir et al., 2006 (Weir et al., 2006); ^b^Sandborn et al., 2004 (Sandborn and Faubion, 2004); ^c^Tracey et al., 2008 (Tracey et al., 2008); ^d^Sedger et al., 2014 (Sedger and McDermott, 2014); ^e^Scallon et al., 2002 (Scallon et al., 2002); ^f^Mitoma et al., 2018 (Mitoma et al., 2018); ^g^Shealy et al., 2010 (Shealy et al., 2010); ^h i^Atzeni et al., 2012 (Atzeni et al., 2012); ^j^Lanternier et al., 2013 (Lanternier et al., 2013); ^k^Curtis et al., 2011 (Curtis et al., 2011); ^l^Chiang et al., 2014 (Chiang et al., 2014); ^m^Curtis et al., 2012 (Curtis et al., 2012), ^n^Wallis et al., 2004 (Wallis et al., 2004). For further information see Mitoma et al., *Cytokine* 2018 (Mitoma et al., 2018). + is the relative risk of infection where ++ is a greater risk than +, and +++ is a greater risk than ++. N/A, Not available.

### 2.1 Structure of TNF inhibitors

The structure of TNF inhibitors plays a key role in their effects. Infliximab, adalimumab, and golimumab are all IgG monoclonal antibodies (mAb), with infliximab being a chimeric antibody with a murine variable region and human Fc region, and the latter two being fully-humanized IgG mAbs (Mitoma et al., 2018). Etanercept is composed of a human IgG Fc region fused with the extracellular portion of TNF receptor (TNF-R) (Mitoma et al., 2018). Certolizumab is composed of polyethylene glycol covalently attached to a human anti-TNF-α Fab region (Mitoma et al., 2018). While other TNF inhibitors do exist, these are the only ones that have been FDA-approved at the time of the writing of this review. These different structures result in differential binding and inhibition potential between each drug ultimately leading to varying risks and efficacies for treating each disease (Sandborn et al., 2007; Da et al., 2013; Levin et al., 2016; Murdaca et al., 2019; Caporali et al., 2021). Mitoma et al. theorized that the differences in binding affinities and specificities may in part be due to stoichiometric or dosage differences, rather than affinity (Mitoma et al., 2018). For example, Infliximab (mAb-based) binds to both the monomeric and trimeric forms of TNF-α, while etanercept (TNF-R-based) loosely binds to TNF-α solely in the homotrimeric form (Scallon et al., 2002). Presumably, the other mAb-based TNF inhibitors share this ability to bind both monomeric and trimeric TNF-α.

### 2.2 Effects of TNF inhibitors

**Figure 1:**
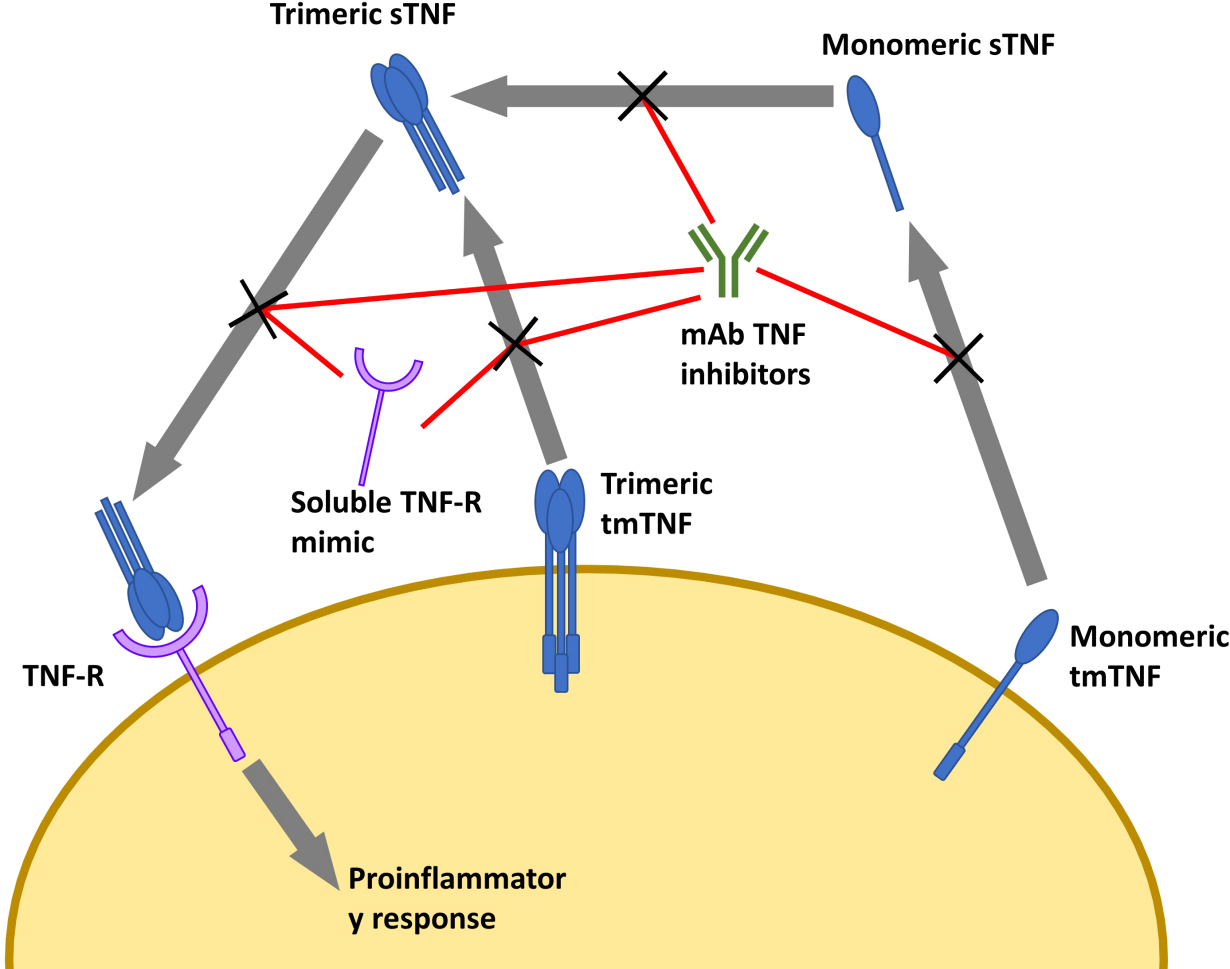
Overview of TNF inhibitors and their targets. Cell depicted is a macrophage. Soluble TNF-R mimic is etanercept. mAb TNF inhibitors include infliximab, adalimumab, golimumab, and certolizumab (While not a mAb, certolizumab is functionally similar). sTNF, soluble TNF; tmTNF, transmembrane TNF; TNF-R, TNF receptor; mAb, monoclonal antibody.

Several studies found that the binding affinity and neutralization ability of different TNF inhibitors are comparable. However, the biophysical properties do not fully explain the differences seen in their resultant infection risks (Nesbitt et al., 2007; Kaymakcalan et al., 2009; Shealy et al., 2010; Murdaca et al., 2019). Despite targeting the same pathway, the molecular targets and subsequent effects differ (**Figure 1**). mAb-based TNF inhibitors are capable of binding two transmembrane TNF-α (tmTNF) or soluble TNF-α (sTNF) molecules in both monomeric and trimeric form (**Figure 1**) (Scallon et al., 2002). Conversely, etanercept can only bind to trimeric TNF-α, but its ability to bind tmTNF is relatively poor (Scallon et al., 2002). As a result, the molar potency of mAb-based inhibitors is higher than that of etanercept, theoretically resulting in a higher degree of immunosuppression and thus, higher propensity to develop infectious complications (Tubach et al., 2006; Lanternier et al., 2013; Padmapriydarsini et al., 2021). Some exceptions do occur, as immunosuppressors have been shown to reduce the magnitude of the cytokine release syndrome in infections with pathogens that rely on it as part of their pathogenesis, improving prognosis (Wright et al., 2004; Ablamunits and Lepsy, 2022). There is also a significant difference in the frequency of infections in individuals who use etanercept compared to those taking mAb-based treatments (Wallis et al., 2004).While anti-TNF-α mAbs exclusively bind to TNF-α, etanercept’s TNF-R domain also binds to lymphotoxin-α (otherwise known as TNF-β), reducing the etanercept available that can bind TNF-α (Cope et al., 1992; Schneider et al., 2004; Murdaca et al., 2012). Compared to etanercept, infliximab also forms more stable complexes with lower rates of dissociation, which may explain the differences in therapeutic and adverse effects (Scallon et al., 2002). Because of the biological roles of mAb Fab regions and TNF-Rs dictating the stability of their binding to TNF, it can be hypothesized that other anti-TNF-mAb’s would produce similar effects to infliximab. However, this is ultimately dependent on the targeted epitope, affinity, and the resultant neutralization capacity (Mitoma et al., 2018). TNF inhibitors also differ in the conditions that could be treated. For example, etanercept has been found to not induce endoscopic remission in IBD, while the anti-TNF-α mAb’s do (Nesbitt et al., 2007; Levin et al., 2016; Murdaca et al., 2019; Caporali et al., 2021). Furthermore, etanercept is also ineffective in treating granulomatous inflammatory conditions (Wallis et al., 2004; Biancheri et al., 2015).

Variabilities in efficacy and safety of TNF inhibitors may be in part due to their half-lives and the effects that are maintained upon cleavage. TNF inhibitors are susceptible to cleavage by proteases. Biancheri et al. reported that infliximab and adalimumab are degraded into an Fab and an Fc region, allowing for them to continue to neutralize TNF-α with their Fab regions. Certolizumab is unique in that it has no effector functions due to the absence of an Fc domain, making neutralization its sole function (Tracey et al., 2008). In contrast to mAb-based inhibitors, etanercept’s TNF-R domain loses its function after proteolytic cleavage, neutralizing its effects (Biancheri et al., 2015). Another possible explanation for the differential infection risks is that a mechanism independent of TNF-α neutralization is responsible in some of the mAb’s. However, this has not been shown to be the case, thus far.

The previous discussion looked at TNF-α as a ligand, but TNF-α may act as a receptor when an agonist binds to tmTNF-α. This binding results in outside to inside signaling, inducing apoptosis of that cell (Kirchner et al., 2004; Ringheanu et al., 2004; Mitoma et al., 2005; Shen et al., 2005). In contrast to the mAb therapies, etanercept is unable to activate outside to inside signaling, consequently leading to less immunosuppression compared to anti-TNF-α mAb’s (Scallon et al., 2002; Kirchner et al., 2004; Horiuchi et al., 2010).

Ultimately, differences in the structure, biochemistry, and biophysics impact the interactions made between TNF inhibitors and their physiological targets, dictating the resultant effects of each agent.

## 3 Effects of existing and potential immunomodulators on pneumonia susceptibility and severity

In this section the mechanisms by which TNF inhibitors and other immunomodulators predispose or protect individuals from infection by select respiratory pathogens will be explored. The immunomodulators discussed in this section will include TNF-α (and TNF inhibitors), metformin, and various cytokines/cytokine mimics, and cytokine antagonists.

### 3.1 Mycobacterium tuberculosis

*M. tuberculosis* infections can manifest across a spectrum of disease spanning from asymptomatic latency to severe pneumonia that can prove lethal if left untreated. Unlike traditional bacteria, members of the *Mycobacterium* genus have a unique cell wall composition that includes mycolic acid, arabinoglycan, and a modified muramic acid in addition to the traditional cell wall constituents such as N-acetyl glucosamine (Lederer et al., 1975; Mahapatra et al., 2000; Mahapatra et al., 2005; Raymond et al., 2005). This causes the cell wall to be more resistant to traditional antibiotics, requiring specific anti-tuberculin treatments for the infection. *M. tuberculosis’* ability to occupy hosts in a state of latent or incipient TB makes administration of immunosuppressing agents difficult due to the risks of reactivation. These risks become even more pronounced as the world becomes increasingly connected and individuals emigrate from TB endemic areas. Thus, the infection risks associated with specific immunosuppressors must be evaluated.

#### 3.1.1 TNF-α and TNF inhibitors

To avoid activation or reactivation of *M. tuberculosis* infection, individuals are screened and given prophylactic treatment when possible, prior to starting TNF inhibitor therapies (Carmona et al., 2005; Chen et al., 2008; Goletti et al., 2018). As stated by Kaptan et al., “… different risk factors have been found in different studies and more data are clearly needed to define high-risk subgroups for whom alternative preventative approaches may be developed” (Kaptan et al., 2021).

Granulomas are characterized by the recruitment of immune cells to a site of infection, where the immune cells surround the infected cells and can suppress the spread of the pathogen while being incapable of eliminating the infection. This creates an environment that needs to be finely maintained, otherwise the pathogen can overcome host defenses to develop into an active infection. Granuloma formation is a key factor in long-lasting tuberculosis due to it providing a haven to hide from the immune system and slowly disseminate new *M. tuberculosis* cells such as in latent TB infection (Adams, 1976; Sandor, 2003). Blocking TNF-α signaling disrupts the formation of this granuloma by reducing proinflammatory signals, leading to apoptosis of immune cells maintaining this granuloma, thus allowing the entrapped *M. tuberculosis* to escape and cause active tuberculosis (Jo et al., 2007; Kirschner et al., 2010; Cooper et al., 2011). More specifically, blockage of TNF-α interferes with the maturation and activation of macrophages, dendritic cells, and T cells that participate in granuloma formation and maintenance by attempting to eliminate free pathogens and infected cells (Trevejo et al., 2001; Mehta et al., 2018).

The synergistic effects of TNF-α and IFN-γ are essential in controlling *M. tuberculosis* infections (Xu et al., 2014; Rai et al., 2016). TNF-α is required to maintain granuloma formation and prevent bacterial leakage, while IFN-γ promotes macrophage activation and cytotoxicity (Cooper et al., 1993; Flynn et al., 1993; Mohan et al., 2001; Cavalcanti et al., 2012). When taken in conjunction, these processes are essential for granuloma maintenance, and thus controlling *M.* tuberculosis infections. In an *in vitro* study, the *M. tuberculosis* protein Rv0309 has been shown to inhibit the production of TNF-α in addition to IL-6 and IL-1β (Peng et al., 2022). Kamboj et al. found that PD-1 inhibition restored proinflammatory cytokines TNF-α and IFN-γ to rescue macrophages infected *in vitro* and in mouse models. However, the ability of PD-1 inhibition to rescue macrophages in a human granuloma remains to be seen (Kamboj et al., 2020).

Between 1998 and 2002, the US Food and Drug Administration’s Adverse Event Reporting System received significantly more reports from patients, healthcare professionals, and pharmaceutical companies of individuals being infected with *M. tuberculosis* while taking infliximab compared to those taking etanercept, despite similar numbers of individuals taking both types of drugs for rheumatoid arthritis (Keane et al., 2001; Mohan et al., 2004). In a 2004 investigation into adverse effects associated with infliximab and etanercept, *M. tuberculosis* was observed to be the most frequent cause of infections, but a later study looking at non-viral infections across all TNF inhibitors found that it had dropped to third place (59% to 12.5%) (Wallis et al., 2004; Baddley et al., 2014). In the former study, it was found that infliximab was responsible for roughly 3 times as many infections as etanercept (Wallis et al., 2004). Despite screening and treating for TB infection prior to TNF inhibitor therapy, it remains a frequent cause of pneumonia after starting TNF inhibitor therapy (Borekci et al., 2015; Cagatay et al., 2018; Kaptan et al., 2021). While guidelines often only recommend screening for TB infection prior to TNF inhibitor therapy, these studies have shown that TNF inhibitors not only reactivate TB infection, but also increase susceptibility to new infections, stressing the importance of thorough surveillance of infection throughout treatment.

Overall, the choice of TNF inhibiting agent has been shown to impact susceptibility to *M. tuberculosis* infection by interfering with granuloma formation (Alves da Silva et al., 2018; Mezouar et al., 2019). Supplementation of TNF-α and IFN-γ may attenuate the TNF inhibitor-induced resolution of granulomas. Further studies need to be done *in vivo* to evaluate the effectiveness of partial TNF-α and IFN-γ restoration by supplementation in clearing infection rather than exclusively to maintain the granuloma. While supplementation may seem counterintuitive, partial restoration of TNF-α and IFN-γ may allow for infection clearance without amplifying the immune response enough to cause sequelae.

#### 3.1.2 Metformin

Metformin has been seen to have context-dependent immunosuppressive and immunostimulatory effects, acting as an immunosuppressant in infections and an immunostimulant when accumulated in tumour cells (Marcucci et al., 2020). Marcucci et al., theorize that high concentrations of metformin may have an immunostimulatory effect (such as in tumour cells, where it is allowed to accumulate) and low concentrations have an immunosuppressive effect (Marcucci et al., 2020). Evidence exists for metformin use in treatment of both tuberculosis and rheumatological diseases, suggesting that metformin may even be a safer alternative to TNF inhibitors (Gharib et al., 2021; Kim et al., 2022). In addition, metformin also facilitates phagolysosome fusion in macrophages indicating that there is further potential for metformin to aid infections caused by intracellular pathogens, such as *M. tuberculosis* (Singhal et al., 2014; Wallis and Hafner, 2015; Rana et al., 2021; Bone et al., 2021). Metformin’s protective effects against several pathogens is widely believed to be caused by its ability to activate AMPK, upregulating mitochondrial reactive oxygen species (mROS) production and activating autophagy (West et al., 2011; Singhal et al., 2014; Wallis and Hafner, 2015; Kajiwara et al., 2018). However, metformin has also been shown to inhibit the oxidative phosphorylation pathway and mTOR, a protein involved in processes such as stress responses, metabolism, cell growth, and autophagy (Ravikumar et al., 2004; Mohammed et al., 2013; Bridges et al., 2014). These factors all play an important part in the early immune response, making metformin a contender for immunotherapy.

Padmapriydarsini et al., found conflicting results when comparing people affected by TB who were administered a combination of metformin and rifampicin (METRIF) with those that were administered rifampicin without metformin. While it was found that METRIF did not improve sputum culture conversion, it did reduce chest cavities and inflammatory markers (Padmapriydarsini et al., 2021). Similar findings were observed by Lee et al. (2018). The reduction in inflammatory markers could be argued to be a result of metformin’s role as an immunosuppressor. Another study by Heo et al., found that individuals who received larger cumulative doses of metformin over time prior to being infected with *M. tuberculosis* experienced a therapeutic effect (Heo et al., 2021). The dosage per treatment, however, was not stated nor examined and the study did not follow the cohort throughout the treatment. These findings complemented by a study from Dutta et al. that found that metformin did not offer additional preventative effects when paired with anti-tuberculosis treatment in mice until they had been on metformin for 3.5 months (Dutta et al., 2017). Contrarily, Singhal et al. found that metformin did reduce *M. tuberculosis* colony-forming units (CFU) in mice when provided as an adjuvant by facilitating phagolysosome fusion (Singhal et al., 2014). The differences in these findings may be a result of Dutta et al. treating mice with an anti-tuberculosis combination therapy while Singhal et al. used metformin along with a single anti-tuberculosis agent (Singhal et al., 2014; Dutta et al., 2017). Heo et al. theorized that early metformin treatment either 1) downregulates TNF-α and IFN-γ, or 2) suppresses anti-tuberculosis agents (Lin et al., 2007; Hyun et al., 2013; Singhal et al., 2014; Caporali et al., 2021; Heo et al., 2021).

Notably, later phases of metformin treatment may induce activation of macrophage activity, facilitating mROS production and phagocytosis (Singhal et al., 2014). Further reinforcement of metformin’s prophylactic use in preventing TB infections is found in a study showing that elderly individuals affected by diabetes mellitus had reduced rates of latent tuberculosis infection (LTBI) when taking metformin (Huang et al., 2021). LTBI has been found to be twice as prevalent in individuals with diabetes mellitus compared to those without (Barron et al., 2018; Lin et al., 2019). In addition, old age and diabetes mellitus are both risk factors for contracting TB and even worse infection outcomes (Lee et al., 2016; Restrepo, 2018; Liu et al., 2020). This suggests a possible role for metformin in protection against LTBI.

Lee et al. and Padmapriydarsini et al. propose metformin as a candidate for managing *M. tuberculosis-*induced sequelae, but Lee et al. believe that it is unsuitable as a general adjuvant in people affected by diabetes mellitus (Lee et al., 2018; Padmapriydarsini et al., 2021). More research is needed in elucidating metformin’s possible roles in different phases of infection to determine if it has any merit in either suppressing or curing TB or in reducing sequelae.

#### 3.1.3 Cytokines

While TNF-α and IFN-γ supplementation enhances protection against *M. tuberculosis*, neither are effective in the absence of the other. Kamboj et al. showed that the two work synergistically to offer a protective effect by blocking PD-1, shifting the immune response to favour Th1 responses (IFN-γ, TNF-α) over Th2-dominated responses (IL-10, TGF-β) (Kamboj et al., 2020). By challenging mice with recombinant Rv0569, a *M. tuberculosis* latency protein, prior to infection, Kanaparthi et al. observed that *M. tuberculosis* clearance was enhanced (Kanaparthi et al., 2022). This clearance was associated with a cytokine milieu favouring Th1 responses (IL-12p40, TNF-α, and IFN-γ) (Kanaparthi et al., 2022). Taken together, both studies indicate that there is a benefit in administering interleukins that promote proinflammatory Th1-dominant responses; IFN-γ, and IL-12p40, while dissuading anti-inflammatory Th2 response by inhibiting IL-10 and TGF-β signaling. Two avenues of approach can be taken in order to achieve this: 1) supplementing Th1 responses by administering IL-12p40, TNF-α, and IFN-γ or mimics of these cytokines, or 2) inhibition of Th2 responses *via* IL-10 and TGF-β blockade in a manner similar to TNF inhibitors.

### 3.2 Legionella pneumophila

*Legionella* is the causative agent of Pontiac fever, a self-limiting flu-like illness; and Legionnaires’ Disease (LD), a severe form of pneumonia that may last several months and lead to mortality (Government of Canada, 2019). In 2020, 295 individuals were diagnosed with LD in Ontario, Canada (Public Health Ontario). Approximately 80% of these patients were hospitalized, and among these individuals there was a 10% mortality rate (Public Health Ontario). *Legionella* is notoriously spread through man-made water systems including showers, fountains, water towers. However, other routes of spread have been documented. *Legionella longbeachae* is spread through soil, and *Legionella pneumophila* has been reported to be capable of human-to-human transmission (Currie and Beattie, 2015; Correia et al., 2016).

In recent years, *Legionella* spp. have been shown to be increasing in incidence (Barskey et al., 2022). Recent findings from our group have shown that the distribution of legionella serogroups varies greatly between geographic regions and the prevalence of other serogroups may be underestimated due to the widespread clinical use of urinary antigen tests in diagnosis, which selectively targets *Legionella pneumophila* serogroup 1 (Head et al., 2019). A study by Head et al. had shown that non-*pneumophila* serogroup 1 *Legionella* (including *L. bozemanae*, *L. micdadei*, *L. anisa*, and other strains of *L. pneumophila*) were found to increase mortality while also doubling the likelihood of requiring intensive care unit (ICU) care in individuals coinfected with HIV and *M. tuberculosis* or HIV and *P. jirovecii* (Head et al., 2019). As reported by the CDC, cases of *Legionella* in the US have been increasing significantly over the past 20 years (Barskey et al., 2022). A similar trend had also been noted in France by Campese et al., following the establishment of mandatory reporting of *Legionella* and stricter detection protocols (Campese et al., 2011). It is unclear whether this reflects an increase in prevalence or increasingly rigorous diagnostic procedures.

Despite most *Legionella* infections being undetected, they are treatable. Currently, effective treatments for *Legionella* infections are limited to macrolides, fluoroquinolones, and glycylcyclines (Muder and Yu, 2002; Arget et al., 2019). The lack of antibiotics available to treat *Legionella* infections combined with the need to reduce the antibiotic footprint in the healthcare system highlights the importance of discovering alternative treatments that can be used to treat *Legionella* infections. Immunomodulators may be fit to fill this role for two reasons: 1) their ability to keep infections under control and improve clinical outcomes, and 2) the pre-existing approval and existence of these drugs, bypassing the early stages of preclinical studies and phase 1 drug trials necessary to prove their safety and quickening the pace of transitioning into adjuvants. A majority of studies on *Legionella* focus on *Legionella pneumophila*, which is considered the most virulent *Legionella* and thus will be the topic of focus in this section.

#### 3.2.1 TNF-α and TNF inhibitors

Two surveys from France by Tubach et al. and Lanternier et al. had shown that individuals taking TNF inhibitors had increased susceptibility to *L. pneumophila* serogroup 1 infections, at 13-21 times more likely than the general population (Tubach et al., 2006; Lanternier et al., 2013). Among these individuals, etanercept had the lowest standardized incidence ratio, followed by infliximab, then adalimumab (3.0, 15.3, and 37.7, respectively) (Lanternier et al., 2013). Several of the individuals from this study had also been prescribed corticosteroids. While corticosteroids were not associated with a predisposition to *Legionella* infections, it should be noted that the individuals from this study who had received ICU care had all taken prednisolone or prednisone for other conditions (Stuck et al., 1989; Gotzsche and Johansen, 1998; Doran et al., 2002; Tubach et al., 2006). While Tubach et al. and Lanternier et al. did not consider corticosteroids to be a significant risk factor for legionellosis, the possibility that they may result in exacerbation of disease severity while not affecting susceptibility is not out of the question. It may even be argued that while corticosteroids do not affect susceptibility as the sole treatment, the combination of TNF inhibitors with corticosteroids may worsen the condition. If TNF inhibitors must be used, it is recommended to prescribe etanercept over the monoclonal anti-TNF-α inhibitors whenever possible due to the significantly decreased risk of *L. pneumophila* infection (Tubach et al., 2006; Lanternier et al., 2013).

TNF-α has been shown to protect epithelial cells and macrophages from *L. pneumophila* infection by inducing apoptosis in the early stages of *L. pneumophila* infection in a human airway epithelial cell line (H292) and a monocyte cell line (THP-1) (Skerrett and Martin, 1996; Kawamoto et al., 2017). When supplemented with TNF-a these infected cells were found to increase lactate dehydrogenase and activated caspase 3/7 levels (Skerrett and Martin, 1996; Kawamoto et al., 2017). Multiple studies observed increased levels of TNF-α during infection with various species of *Legionella* (Neumeister et al., 1998; Chang et al., 2004; Kajiwara et al., 2018). Moreover, TNF-α expression was seen to decrease when *L. pneumophila* invasion of A549 cells was inhibited (Chang et al., 2004). Taken together, these data indicate that TNF-α, and thus, macrophage activation, is a necessary step in the immune response against *Legionella* infection.

#### 3.2.2 Metformin

Kajiwara et al. had recently shown that metformin can protect mice and cell lines U937 and RAW 264.7 against *L. pneumophila* infection and even improve prognosis when administered prophylactically, decreasing bacterial load in a dose-dependent manner, but not when administered post-infection (Kajiwara et al., 2018). Kajiwara et al. had also found 5-aminoimidazole-4-carboxamide-1-β-D-ribofyranoside (AICAR), another AMPK activator, to be able to reduce CFU but to a lesser extent than metformin (Kajiwara et al., 2018). Administration of compound C, an AMPK inhibitor, was also observed to reverse this therapeutic effect (Kajiwara et al., 2018). This appears to be the only currently existing study exploring the effects of metformin on *Legionella* infections.

Metformin is also an inhibitor of the NF-κB pathway, which has been shown to be upregulated by LnaB, an effector protein excreted by *L. pneumophila* during infection (Losick et al., 2010). While the increased mROS production is believed to be the cause of protection in cells from intracellular infections, it is plausible that NF-κB inhibition is also an essential step in warding off *L. pneumophila* infections (West et al., 2011; Kajiwara et al., 2018).

One of the primary escape mechanisms employed by *L. pneumophila* is its ability to disrupt phagolysosome fusion, hijacking phagosomes to form a *Legionella*-containing vacuole (LCV). The LCV protects *L. pneumophila* from the immune system and allows it to avoid cellular defenses, while exploiting the host cell’s functions (Roy et al., 1998; Khweek and Amer, 2010; Xu et al., 2010; Xu and Luo, 2013). Similar to what was seen in *M. tuberculosis*, metformin-induced phagolysosome fusion can be expected to bypass *L. pneumophila*’s defense mechanisms (Singhal et al., 2014; Bone et al., 2021; Rana et al., 2021).

While metformin’s effects as a post-infection therapeutic in *L. pneumophila* infections may be lacking in mice, its efficacy remains to be evaluated in humans. At the very least, these studies should be recreated in a humanized mouse model or with adjusted dosages. Although clinical evidence supporting metformin’s protective effects against *L. pneumophila* infections is lacking, there is potential plausible activity suggesting its effectiveness at the cellular level.

#### 3.2.3 Cytokines

IL-1 is a key proinflammatory cytokine, playing a role in the activation of both the innate and adaptive immune system (Shibuya et al., 1998; Ben-Sasson et al., 2009; Sreejit et al., 2020). Asrat et al. has shown that MyD88 plays an important role in bypassing *L. pneumophila*’s inhibition of the immune system by allowing an alternative pathway for proinflammatory cytokine genes such as *tnfα*, *Il1a*, and *Il1b* to be translated (Asrat et al., 2014). Neumeister et al. had also found that IL-1β was upregulated in infections caused by various *Legionella* species, further indicating its importance in infection (Neumeister et al., 1998). In contrast, IL-1β was found by Chang et al. to not be significant in *L. pneumophila* infections, but this may be due to the strain that was used in the study, which was shown to be a possibility by the differences in immune response against different strains of *L. pneumophila* that was observed by Neumeister et al. and Guillemot et al. (Neumeister et al., 1998; Chang et al., 2004; Guillemot et al., 2022). Furthermore, Chang et al. used A549 cells in their study while Neumeister et al. used Mono mac 6, which may be another cause for the differences seen between the two studies (Neumeister et al., 1998; Chang et al., 2004). Supplementation with IL-1 or an activator of MyD88 can thus be hypothesized to stimulate the immune system to counteract the immunosuppression imposed by *L. pneumophila* (Sreejit et al., 2020).

IL-10 induces differentiation of monocytes to M2 macrophages, which carry out an anti-inflammatory role marking the clearance of infection and mediating the switch-off of inflammatory response. Contrarily, IFN-γ induces differentiation of monocytes to M1 macrophages, a key cell in clearing infection. This increase in IL-10 thus serves the double role of inhibiting M1 differentiation and inducing M2 differentiation, allowing the infection to persist. Park & Skerrett found that IFN-γ reduced intracellular growth of *L. pneumophila* in a dose-dependent manner in unstimulated monocytes and alveolar macrophages from healthy volunteers (Park and Skerrett, 1996). Another study by Skerrett et al. expanded on this by examining the synergistic effects of TNF-α and IFN-γ, in which low levels of TNF-α could be supplemented with IFN-γ (Skerrett and Martin, 1996). These findings were synonymous to Kamboj et al.’s study, looking at TNF-α and IFN-γ in *M. tuberculosis* (Kamboj et al., 2020). The same observation was recently made by Maciag et al., who studied the inhibitory effects of IRF3 on IFN-γ in macrophages, creating a permissive environment to encourage the growth of intracellular pathogens (Maciag et al., 2021). Park & Skerrett had also observed worsening infection in monocytes upon treatment with anti-inflammatory IL-10, even reversing the protective effects of IFN-γ (Park and Skerrett, 1996). Kajiwara et al. demonstrated that infection with *L. pneumophila* induced the upregulation of IL-12p35, an anti-inflammatory cytokine inducing IL-10 and IL-35 production in regulatory B cells and regulatory T cells (Dambuza et al., 2017; Kajiwara et al., 2018). With these findings and those of TNF-α’s role in *Legionella* infections, the importance of macrophage activation in the defense against infection by *L. pneumophila* is further reinforced. Altogether, these studies show that there is potential for administration of supplementary IFN-γ and TNF-α, as well as IL-10 and IL-12p35 antagonists to aid in *L. pneumophila* infections. However, IL-12p35 must be further studied due to the role it plays in reducing autoimmunity (Collison et al., 2007; Wang et al., 2014; Dambuza et al., 2017). Yang et al. offers an alternative explanation in which an NF-κB-induced decrease in IFNGR1 rather than the level of IFN-γ expression, creates a permissive state allowing for *L. pneumophila* infection (Yang et al., 2020).

While the importance of macrophage activation can be concluded as being important in controlling *Legionella* infections, the viability of IFN-γ supplementation has yet to be shown in an animal model but shows promise. It is also necessary to determine the limits of IFN-γ supplementation with varying expression levels of IFNGR1.

### 3.3 *Pneumocystis jirovecii* and *Pneumocystis carinii*

*Pneumocystis jirovecii* is an opportunistic fungal pathogen that infects up to 20% of healthy adults in the US in a latent form (U.S. Department of Health & Human Services, 2021). Like *M. tuberculosis*, *P. jirovecii* is also at risk of activation upon immunosuppression or immunocompromisation, necessitating screening prior to administration of TNF inhibitors (U.S. Department of Health & Human Services, 2021). The issue of *P. jirovecii* infections is further complicated upon clearance wherein glucan release is triggered to cause hyperactive immune response-induced sequelae and even mortality (Hoffman et al., 1993; Vassallo et al., 2000; Kottom et al., 2015). Skalski et al. theorizes that the autoimmune damage caused by *P. jirovecii* is reduced in individuals who are immunocompromised, which opens up the possibility for several interesting interventions that may be taken to treat *P. jirovecii* infections such as immunosuppression (Skalski et al., 2015).

#### 3.3.1 TNF-α and TNF inhibitors

In a 2004 study using data collected from the FDA on individuals taking etanercept or infliximab, *Pneumocystis* was not among the top 14 causes of granulomatous infections in the US (n=639) (Wallis et al., 2004). In contrast to the previously mentioned study, a 2014 study also from the US found that *Pneumocystis* was the most common non-viral infection (n=80, 20%) in individuals using TNF inhibitors in general (Baddley et al., 2014). This change in prevalence may be a result of differences in diagnostic practices, a change in prevalence, or advancements in antiretroviral treatments reducing the prevalence of other pathogens. While often co-infected with HIV, it has even been seen in individuals with rheumatic disease and no other predisposing factors (Jacobs et al., 1991; Cano et al., 1993; Smith et al., 1993; Oien et al., 1995). Several studies from Japan have shown that *P. jirovecii pneumonia* risk is increased in individuals on TNF inhibitors (Harigai et al., 2007; Komano et al., 2009; Koike et al., 2012; Tanaka et al., 2012; Watanabe et al., 2012; Koike et al., 2014). A Spanish study had also found that TNF inhibitors increased susceptibility to *P. jirovecii* infection (Wissmann et al., 2011). However, *P. jirovecii* was found to be less common among TNF inhibitor users in the United States (Wallis et al., 2004; Baddley et al., 2014; Grubbs and Baddley, 2014). The differences seen in prevalence may be due to geographical features or even monitoring practices as Japan employs a more rigorous screening process involving PCR, while North America uses sputum samples, bronchial alveolar lavage, or biopsies (Koike et al., 2007; Grubbs and Baddley, 2014; Ontario Agency for Health Protection and Promotion, 2020; U.S. Department of Health & Human Services, 2021). The different mortality rates observed in the two countries is theorized by Grubbs et al. to be due to PCR sensitivity being more strongly influenced by factors such as immunosuppression than *P. jirovecii* presence (Morris et al., 2004; Vidal et al., 2006; Curtis et al., 2012; Grubbs and Baddley, 2014).

Nandakumar et al. found that adoptive transfer of M1 and M2 macrophages into *P. jirovecii-*infected rats improved clearance. While it was concluded that M2 polarization may be more suitable in *P. jirovecii* infection, the experiments involved adoptive transfer of M2 macrophages into rats 8 weeks post-infection instead of inducing differentiation with cytokine supplementation (Nandakumar et al., 2017). M1 macrophages were found to be present after adoptive transfer, indicating that M1 differentiation still occurred as there was no artificial alteration of the differentiation factors (Nandakumar et al., 2017). M1-dominant and M2-dominant responses are representative of *P. jirovecii*-induced immune responses in immunosuppressed and immunocompetent hosts, respectively (Nandakumar et al., 2017). Furthermore, the M2 response was seen to result in reduced inflammation, which is valuable in suppressing sequelae caused by glucan release from dying fungi in the later stages of infection (Nandakumar et al., 2017). Thus, while Nandakumar et al. concluded that induction of M2 polarized macrophages may be more beneficial than M1, the optimal response likely involves a mix of both M1 and M2 macrophages with a temporal separation between the two responses.

By administering IFN-γ, the M1 macrophage response can be stimulated to enhance fungal clearance. Because clearance may cause β-glucan-induced sequelae, there appears to be a benefit in switching individuals over from an M1 to M2 macrophage response later on in infection. Some potential methods to achieve this that could be explored are by inhibiting or supplementing IFN-γ and IL-4 at the appropriate points in infection. TNF inhibitor use may even aid in the reduction of *P. jirovecii*-associated tissue damage caused by an overactive immune response (Vos et al., 2011; Kratochvill et al., 2015). This would be especially important later in infection, as observed by Nandakumar et al. (2017). It is important to note that while TNF inhibitor use may aid in recovery from *P. jirovecii* infections, it does not mean that individuals taking TNF inhibitors are not predisposed to secondary infections or even reinfection. Altogether, these findings appear to agree with Skalski et al.’s theory that immunosuppression may be important in controlling *P. jirovecii* infections (Skalski et al., 2015).

#### 3.3.2 Metformin

Currently, there are no studies looking at the effects of metformin on *P. jirovecii* infection. However, inferences can be made from the results of Nandakumar et al.’s study regarding the adjusted susceptibility. The differences in M1 and M2 macrophage clearing appears to be, in part, derived from the differential reactive oxygen species (ROS) and reactive nitrogen species (RNS) production (Lasbury et al., 2011). M1’s increased killing potential was also seen to be dependent on increased RNS production relative to M2 macrophages (Skerrett and Martin, 1996; Nandakumar et al., 2017). It may then be hypothesize that metformin’s induction of increased mROS production may aid in clearance by compensating for the decrease in RNS (West et al., 2011; Singhal et al., 2014; Nandakumar et al., 2017). However, it may be argued that mROS is not a viable substitute for RNS. Furthermore, metformin can act as an immunosuppressor, which can be expected to aid in severe sequelae.

β-glucan’s ability to trigger a hyperactive immune response has been attributed to NF-κB overstimulation (Evans et al., 2005). In addition to metformin’s aforementioned effects, its function as an NF-κB inhibitor, allows for it to reduce *P. jirovecii*-induced sequelae (Hyun et al., 2013; Besli et al., 2020).

The effects of metformin on severe *P. jirovecii* infection must be further studied, as it is unclear what interactions may occur in the host and how the resultant interactions affect the infection. However, if metformin induces further killing, it is expected that β-glucan release upon fungal death can lead to further complications that may also be suppressed by metformin’s immunosuppressive effects.

#### Cytokines

Several genes were found by Kottom et al. to have significantly altered levels of transcription when infected with *P. jirovecii* (Kottom et al., 2022). Among this list, the majority of genes were significantly increased while only 2 decreased in expression. Despite the size of this list, Alshahrani et al.’s study on mRNA expression in individuals infected with *P. jirovecii* yielded no cytokines in common with Kottom et al. (Alshahrani et al., 2021; Kottom et al., 2022). Alshahrani et al. found that infection by *P. jirovecii* caused an increase in anti-inflammatory factors IL-2, IL-4, IL-10, and IL-13 mRNA (Alshahrani et al., 2021). While these transcripts are produced, it cannot be concluded that a proportional translation occurs. It is not known if their transcription is host-induced as a means of combating the infection or if it is induced by *P. jirovecii* as an escape mechanism. It is also unknown whether the observed cytokine expressions impact the outcomes or if they occurred as a result of the infection. *Il4* and *Il10* gene transcription were found to be associated with worse symptoms and disease while *Il13* transcription was associated with milder cases consisting of only fever (Alshahrani et al., 2021). Higher *Il2* mRNA levels were found to be associated with more *P. jirovecii-*positive cases than *P. jirovecii-*negative cases. These positive cases also experienced milder symptoms. IL-4, IL-10, and IL-13 are known to induce a Th2-dominated response consisting of B cell activation, phagocyte deactivation, and anti-inflammation (Chen et al., 1992; Paul, 2015; Wójtowicz et al., 2019). This is analogous to the M1 and M2 macrophage differentiation previously induced by Nandakumar et al. (2017). In contrast to Nandakumar et al.’s findings in their rat model, Wójtowicz et al. observed worsened outcomes in mice containing a single nucleotide polymorphism (SNP) associated with increased expression of IL-4, which reduces Th1 responses (Akkad et al., 2007; Imran et al., 2012; Li et al., 2014; Anovazzi et al., 2017; Wójtowicz et al., 2019). While these differences may have been due to the different host organisms under study or the study design itself, other studies found that the presence of this SNP increased susceptibility to other fungal infections in humans, including candidiasis in Latvian women and paracoccidioidomycosis in Brazilian individuals, as well as other mouse model studies (Kolls et al., 1999; Babula et al., 2005; Bozzi et al., 2009).

While traditionally considered to be a proinflammatory marker, more recent findings have shown IL-2 to promote Treg development, paradoxically suppressing the immune system (Mingari et al., 1984; Montagnoli et al., 2002; Malek, 2003; Ye et al., 2018). While it is uncertain what IL-2’s role in *P. jirovecii* infections is, both proinflammatory and anti-inflammatory effects have been shown to be protective. IL-2 also induces B cell proliferation, allowing for antibody-mediated neutralization of glucans to reduce the magnitude of glucan-induced cytokine release syndrome (Mingari et al., 1984; Montagnoli et al., 2002; Torosantucci et al., 2009). We propose that anti-inflammatory factors be inhibited until the later stages of infection in which the fungi are nearly cleared then switching to supplementing anti-inflammatory factors to minimize damage. Ultimately, these works indicate that while it is important to balance M1/Th1 and M2/Th2 responses, the timing of the responses may be vital to minimizing the overall damage caused by *P. jirovecii* infection.

**Figure 2:**
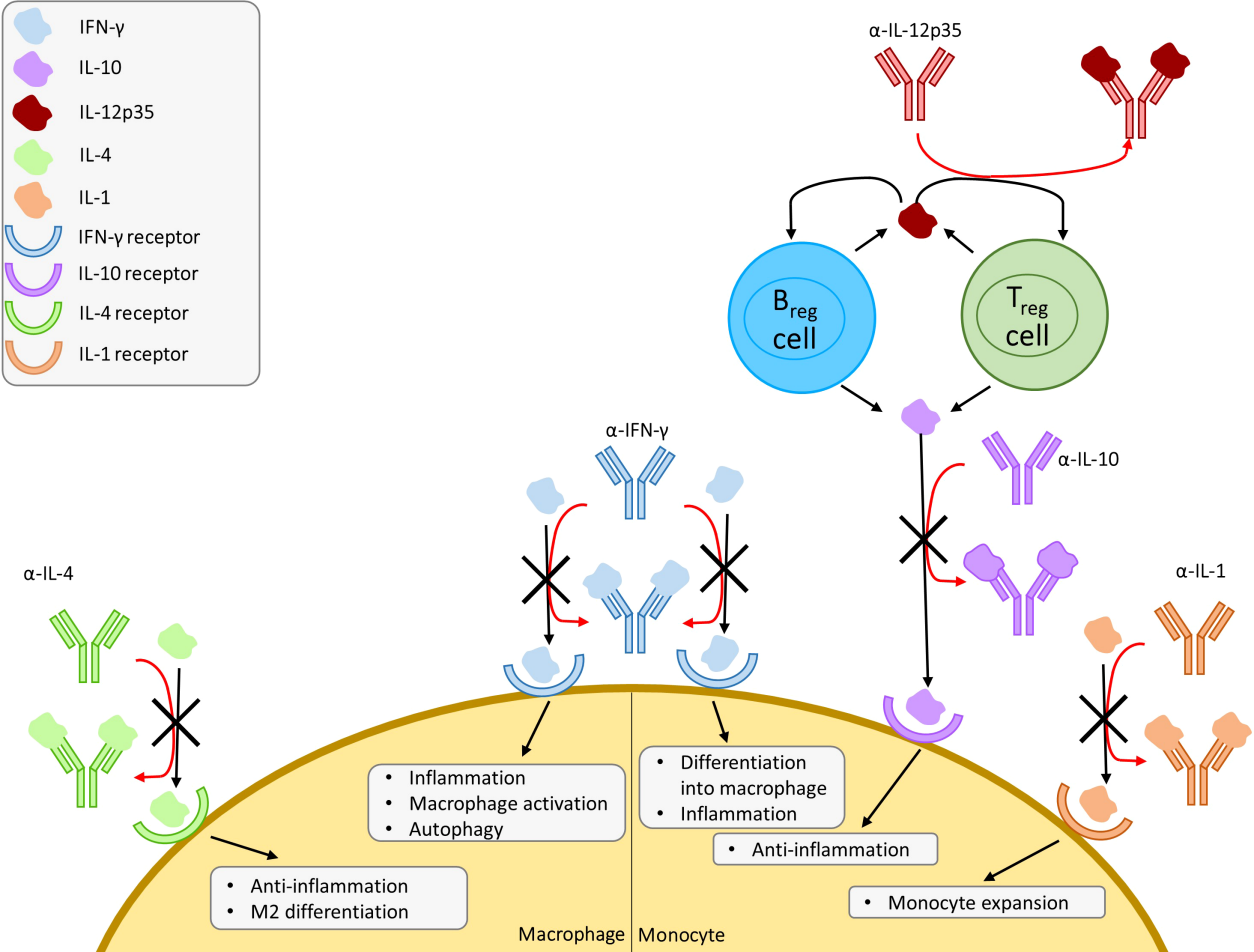
Overview of proposed cytokine mimic and antagonist interventions with a focus on macrophages and monocytes. Proposed mechanism of induced susceptibility and resistance against infection in macrophages. Regulatory T cells (Treg) and regulatory B cells (Breg) produce IL-12p35, which promote IL-10 production in Treg and Breg. IFN, interferon; IL, interleukin.

The drugs and proposed treatments covered in this section are all host-targeted treatments that modulate the innate immune system with an emphasis on macrophage activation or deactivation, with the exception of metformin, which has paradoxical effects as both an immunosuppressor and immunostimulant (Marcucci et al., 2020). The studies that have been reviewed indicate that non-antibiotic treatments have potential for controlling infections caused by *M. tuberculosis*, *L. pneumophila*, and *P. jirovecii* but more research is needed. The summarized pathways and proposed interventions can be found in **Figure 2**.

## 4 Perspectives

TNF inhibitors increase susceptibility to infections, especially those attributed to intracellular pulmonary pathogens. The spectrum of associated causative agents for pneumonia and relative risk of infections varies and is often confounded by parallel or sequential immunomodulatory agents administered. Each subclass has distinct characteristics and associated risks that seem to be influenced by their binding partners and the nature of the interaction. The mAb-based TNF inhibitors can be used to treat and modify the course of inflammatory conditions but cause a heightened risk of infection compared to the TNF-R-based counterpart. Evaluation of the patients’ comorbidities and risk for infection are critical to guide the choice of alternate immune modulators and determine the need to screen for latent infections as well as follow-up after initiation of therapy. *P. jirovecii* is present in a significant portion of the world’s population as latent infections, making them a concern when prescribing TNF inhibitor therapy. In the case of *M. tuberculosis*, the fact that trials are conducted in high-income countries with low incidence of TB infection obfuscates the risk evaluation when the risks are higher in lower-income countries.

The three pathogens covered in this review primarily infect macrophages, but the pathogeneses are dissimilar, leading to different risks that must be considered when administering host-directed therapies. While this must be approached with caution, it appears to be beneficial to prescribe additional immunomodulators as adjuvants alongside other treatments provided to individuals who are immunosuppressed or immunocompromised. Additionally, like antibiotics, the responses induced by immunomodulators are not universally applicable. There exists potential to exacerbate diseases, as was seen by the proinflammatory M1 macrophage response in *P. jirovecii* infection and TNF inhibitors in general. While immunosuppression is associated with increased infection risk, it can be used to ameliorate infection-induced cytokine release syndromes such as those caused by SARS-CoV-2 or *Staphylococcus aureus* (Iannaccone et al., 2019; Goda et al., 2021; Liao et al., 2021; Ablamunits and Lepsy, 2022; Usman et al., 2022).

Narrow therapeutic window and the potential to induce an overreactive immune response or severely crippled immune system dictate cautious consideration of this form of therapy. Within the context of adjuvants used alongside TNF inhibitor treatment, these proposed treatments are meant to aid in infections without triggering an onset of severe inflammation. Outside of individuals using TNF inhibitor treatment there may be room to adjust dosages to modulate the rate and magnitude of inflammation as needed. No previous studies looking at the effects of the combined use of multiple immunomodulators in an attempt to attenuate the risk of infection or damage caused by infection were found in our literature search. Therefore, it is an area that will benefit from further study.

The clearance of all the pathogens that were covered in this review appear to benefit from IFN-γ supplementation. This indicates that while the viability of immunotherapy varies, select immunomodulators may find broad spectrum use, much like antibiotics. Some immunomodulators, particularly cytokine mimics, may even be able to supplement the loss of specific immune cells by replacing the key cytokines produced by the lost cell type. However, this would have limited applications in the loss of cells that directly induce cytotoxic effects such as granulocytes, macrophages, cytotoxic T cells, and natural killer cells. Nandakumar et al.’s study is of particular interest because it showed potential for activated immune cell transplant as a potential therapy for infections that may even extend to individuals who are immunocompromised, addressing the issue of insufficient immune cell counts (Nandakumar et al., 2017).

Of the immunomodulators covered in this review, metformin appears to be of particular interest. Several studies, as reviewed by Malik et al., have shown the protective effects offered by metformin against intracellular respiratory pathogens, both viral and bacterial, that were not mentioned within this review (Malik et al., 2018; Isnard et al., 2020; Mishra et al., 2022; Usman et al., 2022).

Because of the difficulty in conducting studies looking at infections in individuals taking immunomodulatory agents, monitoring tools should be used to aid in both treatment and further studies. By keeping descriptive records of infections among individuals receiving long-term treatments in a surveillance or reporting program, retrospective analyses are more easily conducted, and less prominent unintended effects of drugs can be made clearer. Several of these programs have been used among the studies that have been covered in this review, but many others exist. Among these are REDCap, a web-based service that can be used to build surveys and clinical databases; SECURE-IBD, a surveillance tool for tracking COVID-19 cases in persons living with IBD; AERS, the adverse event reporting system from the US FDA; and RATIO, a French registry collecting data on infections occurring in individuals who use TNF inhibitors. Diligent usage of these tools with special attention to incidence, clinical presentation, and severity of infections, both nationally and internationally will allow better estimation of the risks associated with these immune modulators. Additionally, it also reduces the burden of having to recruit study subjects, allowing for faster data collection and larger sample sizes. This can be especially useful for stage 4 clinical trials and the early stages of pandemics to identify risks or therapeutic candidates. However, while there are many such tools that exist for these uses, the lack of a standard program results in cases being scattered across multiple databases, posing an issue if inconsistencies exist in the types of data that can be inputted.

Overall, TNF inhibitors pose significant risks in infection, with TNF-R mimics posing relatively lower risks compared to α-TNF mAb’s. While etanercept appears to have to impose the lowest infection risk among TNF inhibitors, but as outlined by Gerriets et al., each drug has varying effectiveness against each condition, making it harder to reduce infections in individuals with conditions that are unable to be treated with lower infection risk agents (Wallis et al., 2004; Biancheri et al., 2015; Gerriets et al., 2021). While each agent has different routes of administration, it is unclear how much this impacts infection risks or the effectiveness of these drugs, and this should be further explored (Gerriets et al., 2021). There is also a need for further studies on the infection risks associated with the newer agents, golimumab and certolizumab. The complexity of immunomodulator therapy is multi-faceted, with many considerations obfuscating the choice of agent to minimize risks and maximize benefits. Among these considerations are patient comorbidities, concurrently administered therapies, epidemiological characteristics, risk of exposures, and duration of immunosuppressive therapy. For more robust risk evaluations on the predispositions of infection, studies need to focus on higher risk populations within low- and middle-income countries or diverse populations within high-income countries.

## Author contributions

YK, ZR, and RH defined the review question and the topics to be reviewed. RH draft the manuscript. YK and ZR revised the paper critically for important intellectual content. All authors approved the final version to be published.

## Funding

This manuscript was also supported, in part, by the Canada Research Chairs Program for Dr. Rueda, and through the master’s scholarship for RH. Award number: 323473.

## Acknowledgments

The authors would like to express their gratitude to Dr. Keith Fowke for reviewing the manuscript.

## Conflict of interest

The authors declare that the research was conducted in the absence of any commercial or financial relationships that could be construed as a potential conflict of interest.

## Publisher’s note

All claims expressed in this article are solely those of the authors and do not necessarily represent those of their affiliated organizations, or those of the publisher, the editors and the reviewers. Any product that may be evaluated in this article, or claim that may be made by its manufacturer, is not guaranteed or endorsed by the publisher.
